# PatagoniaMet: A multi-source hydrometeorological dataset for Western Patagonia

**DOI:** 10.1038/s41597-023-02828-2

**Published:** 2024-01-02

**Authors:** Rodrigo Aguayo, Jorge León-Muñoz, Mauricio Aguayo, Oscar Manuel Baez-Villanueva, Mauricio Zambrano-Bigiarini, Alfonso Fernández, Martin Jacques-Coper

**Affiliations:** 1 Facultad de Ciencias Ambientales, Centro EULA-Chile, Universidad de Concepción, Concepción, Chile; 2 Departamento de Química Ambiental, Universidad Católica de la Santísima Concepción, Concepción, Chile; 3 Centro Interdisciplinario para la Investigación Acuícola (INCAR), Concepción-Puerto Montt, Chile; 4 Centro de Energía, Universidad Católica de la Santísima Concepción, Concepcion, Chile; 5 Hydro-Climate Extremes Lab (H-CEL), Ghent University, Ghent, Belgium; 6 Departamento de Ingeniería Civil, Universidad de La Frontera, Temuco, Chile; 7 Center for Climate and Resilience Research (CR2), Santiago, Chile; 8 Departamento de Geografía, Mountain GeoScience Group, Universidad de Concepción, Concepción, Chile; 9 Programa Ciencia Interdisciplinaria para las Montañas de los Andes del Sur (CIMASur), Universidad de Concepción, Concepción, Chile; 10 Departamento de Geofísica, Universidad de Concepción, Concepción, Chile; 11 Center for Oceanographic Research COPAS-Coastal, Universidad de Concepción, Concepción, Chile

**Keywords:** Hydrology, Atmospheric science

## Abstract

Western Patagonia (40–56°S) is a clear example of how the systematic lack of publicly available data and poor quality control protocols have hindered further hydrometeorological studies. To address these limitations, we present PatagoniaMet (PMET), a compilation of ground-based hydrometeorological data (PMET-obs; 1950–2020), and a daily gridded product of precipitation and temperature (PMET-sim; 1980–2020). PMET-obs was developed considering a 4-step quality control process applied to 523 hydrometeorological time series obtained from eight institutions in Chile and Argentina. Following current guidelines for hydrological datasets, several climatic and geographic attributes were derived for each catchment. PMET-sim was developed using statistical bias correction procedures, spatial regression models and hydrological methods, and was compared against other bias-corrected alternatives using hydrological modelling. PMET-sim was able to achieve Kling-Gupta efficiencies greater than 0.7 in 72% of the catchments, while other alternatives exceeded this threshold in only 50% of the catchments. PatagoniaMet represents an important milestone in the availability of hydro-meteorological data that will facilitate new studies in one of the largest freshwater ecosystems in the world.

## Background & Summary

High quality ground-based hydrometeorological observations contribute to the development of high quality policies and management of natural resources^1^. Conversely, unrepresentative, poorly collected, or incorrectly archived data introduce uncertainty into the magnitude, rate, and direction of environmental change, undermining confidence in decision-making processes^2^. The use of hydrometeorological variables is critical in a wide range of environmental, ecological, and hydrological applications. Therefore, there is a need for accurate datasets that include quality-controlled measurements that help address key challenges related to climate change and the impacts of hydrometeorological extremes^3–5^. In addition, hydrometeorological data must be findable, accessible, interoperable and reusable^6^ (FAIR data), requirements that are often not fulfilled in operational datasets^7,8^. Therefore, data obtained from measurements do not always represent the real behaviour of the observed processes^9^, and need to be corrected by homogenisation schemes^10,11^. Furthermore, the generation of large datasets may cross jurisdictions or institutions, lack of common standards or data formats (e.g., quality codes), require manual data collection (e.g., one time series at a time) and are more likely to suffer from spatial and temporal gaps^12^.

The difficulty in accessing ground-based time series has led users to rely on global hydrometeorological datasets, such as reanalyses or satellite products^13–15^. Gridded datasets are very important for deriving regional atmospheric processes for a variety of scientific applications^16^. However, in poorly instrumented areas, gridded datasets can have important biases that need to be corrected^17^ to avoid, for example, systematic underestimation of precipitation in mountainous catchments^18,19^. To address these limitations, different models have used vertical precipitation gradients^20^, precipitation (bias correction) factors^21,22^, or snow correction factors^23,24^. At the global scale, Beck *et al*.^19^ concluded that global products tend to underestimate precipitation (precipitation factors >1.5) over regions characterised by pronounced elevation gradients, low station density, and significant solid precipitation.

The recent development of the Catchment Attributes and Meteorology for Large-sample Studies^25^ (CAMELS) initiative has facilitated the access to hydrometeorological time series and catchment attribute data in the contiguous United States. Since then, similar country-specific datasets have been developed for Chile^18^, Brazil^26^, Australia^27^, Great Britain^28^, China^29^, among others, enabling important advances in large-sample hydrology^12^. These datasets have allowed, among other applications, the estimation of streamflow over ungauged catchments^30,31^, the classification of catchments by hydrological and geomorphological similarities^32^, the impacts related to anthropogenic activities^18,33^, and the analysis of hydrological model structures^34,35^. Despite the existence of the CAMELS initiative for Chile (CAMELS-CL), Western Patagonia is a clear example of how the systematic lack of hydrometeorological data (or open accessibility), poor quality control protocols and multiple formats between institutions^36^ have hindered further studies.

**Fig. 1:**
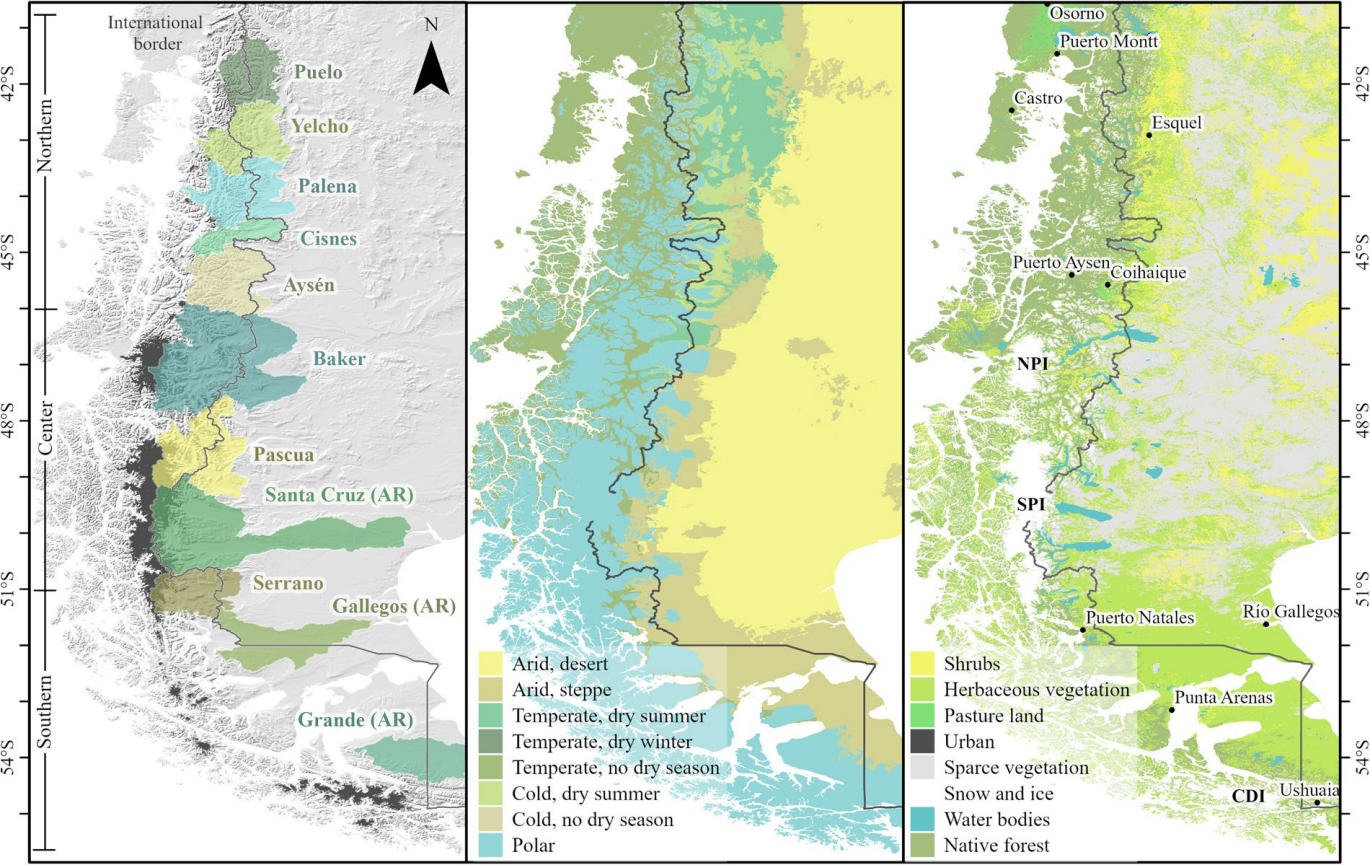
Study area. (**a**) Main basins of western Patagonia (area >5,000 km^2^). AR indicates basins draining to the east. Elevation was obtained from NASADEM^66^. (**b**) Köppen-Geiger climate classification^125^. (**c**) Land cover in the year 2019^126^. NPI: Northern Patagonian Icefield. SPI: Southern Patagonian Icefield. GCN: Gran Campo Nevado. CDI: Cordillera Darwin Icefield.

Western Patagonia is a vast (~400,000 km^2^), narrow (~200–300 km) and transboundary (Chile and Argentina) area extending from about 40°S down to the southern tip of the continent (40–56°S; Fig. 1). It is one of the largest and best preserved freshwater ecosystems in the world, encompassing numerous mountainous catchments of the southern Andes (Fig. 1a), and is surrounded by one of the most complex and extensive fjord systems in the world^37^. Precipitation in this region is mostly generated by disturbances embedded in the westerly flow, with strong orographic gradients^38^. Windward uplift leads to hyper-humid conditions along the western slope of the Andes (>5,000 mm), while downslope subsidence dries the eastern plains, leading to arid conditions^39,40^. The climatic gradients have determined an important climatic spatial variability (Fig. 1b), allowing the development of glaciers, different hydrological regimes^41^ and vegetation types (Fig. 1c).

Climate projections for most of Western Patagonia indicate a prolongation of the dry and warm conditions that have affected it in recent decades^42^. Overall, the climate impacts recorded in Western Patagonia have been attributed to the Southern Annular Mode (SAM), which has shown a significant trend towards its positive phase^43^. Given the heterogeneous and incomplete monitoring network of hydro-meteorological stations, most studies performed in this region have used only a very small subset of meteorological stations^38,44^, satellite imagery^45^ or climate proxies^46^ to study environmental changes. Despite the low use of ground-based information, the region has shown evidence of a decrease in snow cover extent^47,48^, an increase in forest fires^49^, unusual tree growth patterns^50^, a decrease in water availability^51^ and significant trends in major lakes^52^, rivers^41,53^ and glaciers^54,55^.

The low availability of local hydrometeorological data has hindered the development, calibration, and robust validation of regional models for western Patagonia. Krogh *et al*.^56^ implemented the physically-based Cold Regions Hydrological Model (CRHM) in the Baker River Basin (46°S; Fig. 1a) and concluded that the model forced with reanalysis data achieved better performance than the model based on scarce ground-based observations. Recent hydrological modelling efforts have benefited from the integration of local hydrometeorological data with gridded products to achieve better performance^48,53,57^. However, the performance metrics reported by the National Water Balance of Chile using the Variable Infiltration Capacity (VIC) model were not satisfactory in most of western Patagonia^58,59^. Furthermore, the variability of snow accumulation under different atmospheric forcings^39^ has led to divergent surface mass balances in the Patagonian Ice Fields^40,60–62^.

In this study, we present PatagoniaMet (hereafter PMET), a new dataset for Western Patagonia consisting of two datasets: i) PMET-obs, a compilation of quality-controlled ground-based hydrometeorological data, and ii) PMET-sim, a daily gridded product of precipitation, and maximum and minimum temperature. PMET-obs was developed using a 4-step quality control process applied to 523 hydro-meteorological time series (precipitation, air temperature, potential evaporation, streamflow and lake level stations) obtained from eight institutions in Chile and Argentina. In addition, the upstream area corresponding to each stream gauge in PMET-obs was delimited, and climatic and geographic attributes were derived for each catchment. Based on this dataset and currently available uncorrected gridded products, PMET-sim was developed using statistical bias correction procedures, spatial regression models (machine learning) and hydrological methods (Budyko framework). Finally, as part of the validation process, PMET-sim was compared with bias-corrected products using hydrological modelling.

## Methods

### PMET-obs development

The ground-based measurements used to develop PMET-obs were obtained from eight Chilean and Argentinian institutions (see Table 1) for the period 1950−2020, and consist of daily precipitation data (PP), maximum and minimum temperature (Tmax and Tmin, respectively), potential evaporation (Ep), lake/reservoir levels (LL), and streamflow (Q). From the retrieved time series, we selected only those with daily resolution (e.g., the Agrarian Council of the Province of Santa Cruz in Argentina only reports monthly accumulated precipitation), with at least four years of continuous record, and that continue to operate between 2000 and 2020. Regarding the 24-hour period, the data collected correspond to the period from 12:00 to 11:59 UTC, which corresponds to 8:00 to 7:59 (UTC-4) and 9:00 to 8:59 (UTC-3) local time in Chile and Argentina (winter time zone), respectively.

**Table 1:** Data collected by variable, institution and country.

Country	Acronym/Institution		PP	Tmax -Tmin	Ep	Q	LL
Chile	DGA	Dirección General de Aguas	X	X	—	X	X
	DMC	Dirección Meteorológica de Chile	X	X	—	—	—
	ENDESA	Empresa Nacional de Electricidad Sociedad Anónima	—	—	—	X	—
	INIA*	Instituto de Investigaciones Agropecuarias	X	X	X	—	—
	DIRECTEMAR	Dirección General del Territorio Marítimo y de Marina Mercante	—	X	—	—	—
Argentina	RHN	Red Hidrológica Nacional	X	X	—	X	X
	SMN	Servicio Meteorológico Nacional	X	X	—	—	—
	INTA	Red Agrometeorológica	X	X	—	—	—

PP: Precipitation. Tmax and Tmin: Daily maximum and minimum temperature. Q: Streamflow. LL: Lake and reservoir levels. *INIA estimates potential evaporation (Ep) from ground-based observations of wind, relative humidity, solar radiation and air temperature using the Penman-Monteith equation.

**Fig. 2:**
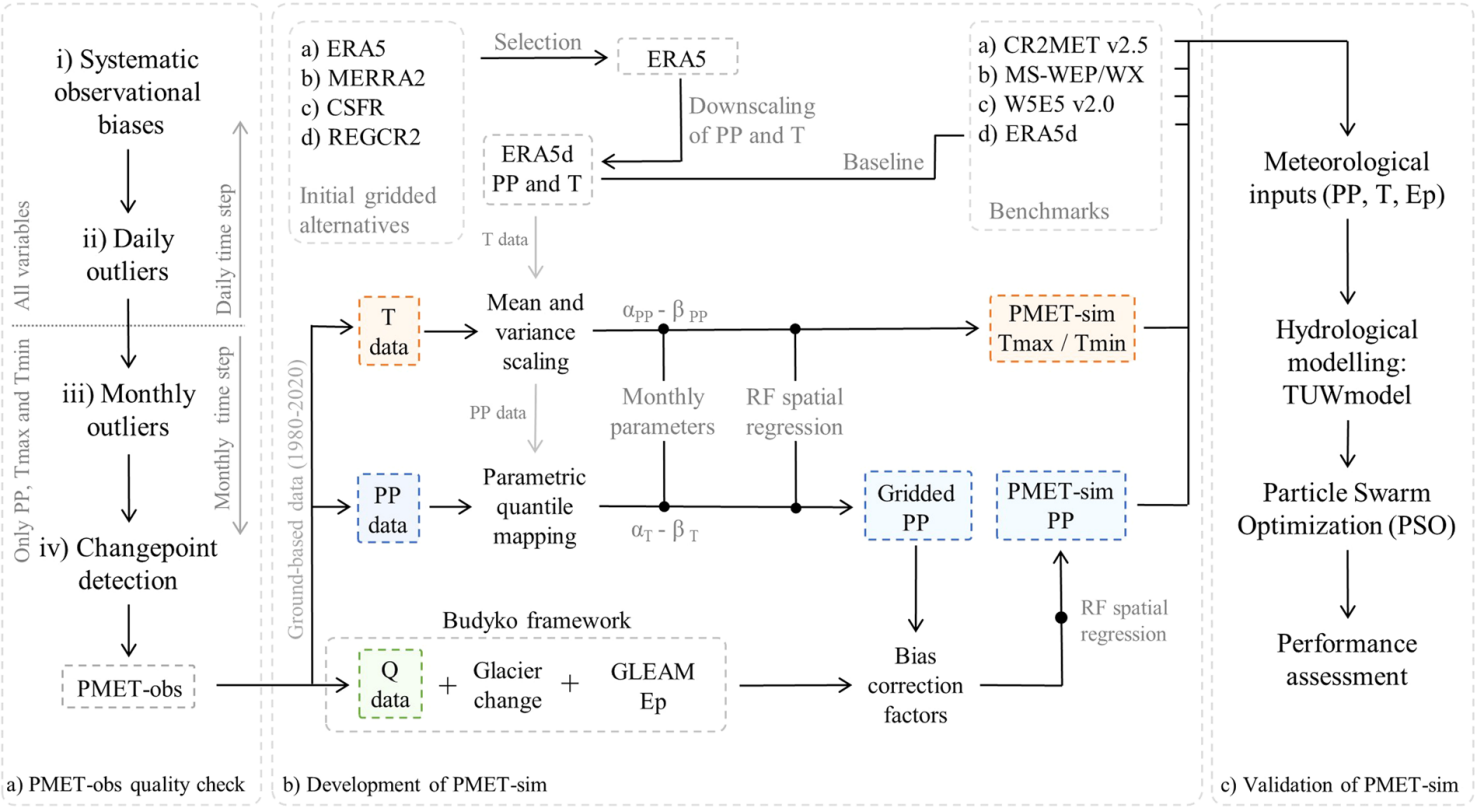
Conceptual methodological framework PMET. (**a**) Quality control of PMET-obs. (**b**) Development of PMET-sim. (**c**) Validation of PMET-sim.

Valuable information on atmospheric and hydrological processes is provided by the eight institutions listed in Table 1. However, these sources of information are subject to various types of disturbance^2^. Although some institutions control the quality of their records, the variety of data management protocols, recording types (automatic vs. manual) and instruments requires a standard quality control to identify and remove anomalous values. We propose a quality control system consisting of four stages, each one associated with a specific objective and time scale (Fig. 2a).

- The first step implemented the recommendations of Wilby *et al*.^2^, who identified several common errors in the information provided by hydrometeorological datasets, such as artificial influences at monitoring sites, changes in reference level, systematic observational biases (e.g., number bias and weekend under-reporting), mislocations, among others. These common errors can skew the observed frequency distribution in ways that may affect the estimation of extreme values and the correct representation of hydrological processes. If the systematic errors were not limited to a specific period, the complete time series was discarded from PMET-obs.
- The second step identified and removed daily outliers from the PMET-obs dataset. These outliers can be attributed to truncation and rounding errors, inconsistent use of missing data flags, suspicious or erroneous data recoded as zero, and data entry for records that were manually digitized. Precipitation outliers were detected using the method proposed by Sarricolea *et al*.^63^ which involves generating reference values from the 10 closest stations within a 125 km radius. We then detected inconsistent temperature measurements (T_max_ < T_min_) or values outside the range of natural variability, i.e., above or below ± 3 standard deviations from the monthly mean. Finally, we detected and removed suspected repeated values in the daily streamflow and lake level time series if the coefficient of variation was less than 0.01 over a one-month window.
- The third step detected and removed monthly residual outliers of the PP, Tmax and Tmin time series using a reanalysis model as a reference. In this step, we selected the ERA5 dataset (Table 2) since it performed better than the other products (see PMET-sim methods) and does not assimilate ground-based data from the study area, as ERA5 only assimilates NCEP Stage IV, which combines NEXRAD data with ground-based precipitation over the conterminous United States. As in the previous step, we eliminated monthly residuals outside the range of natural variability (± 3 standard deviations). In this step, we only considered months with more than 20 days of records.

**Table 2:** Gridded products used during the development (Fig. 2b) or validation of PMET-sim (Fig. 2c).

Product	Variable	Stage	Resolution	Time period	Reference
ERA5	Precipitation and temperature	Development	0.25°	1959 - present	Hersbach *et al*.^67^
MERRA2			0.5°	1980 - present	Gelaro *et al*.^68^
CFSR			0.5°	1979 - present	Saha *et al*.^69^
CR2REG			0.09°	1979 - 2015	Bozkurt *et al*.^70^
CR2MET v2.5		Validation	0.05°	1960 - 2021	Boisier^117^
W5E5 v2.0			0.5°	1979 - 2019	Lange *et al*.^118^
MSWEP v2.8	Precipitation		0.1°	1979 - 2020	Beck *et al*.^14^
MSWX	Temperature		0.1°	1979 - 2022	Beck *et al*.^119^

Temperature includes daily maximum and minimum values.
- The fourth step analysed the existence of (multiple) changepoints in the monthly residuals of PP, Tmax and Tmin, considering ERA5 as a reference. This was done in order to identify potential station relocations or instrumental changes, as this information was not publicly available as part of the metadata for each station. This analysis verified that the mean and variance of the residuals are constant over time, assuming that the equations/parameters that dominate the physics of the reanalysis model are constant over time. The multiple changepoints were identified using the Pruned Exact Linear Time (PELT) algorithm^64^ with a non-parametric cost function based on the empirical distribution of the data. Compared to the traditional parametric approach, non-parametric methods do not assume a particular distribution and are more robust to outliers, which is particularly important considering the probability distribution of precipitation. This algorithm is integrated in the *changepoint.np* R package v1.0^65^, which extends the original *changepoint* package. Once the changepoints were identified, only a subset of the time series was selected based on its extent and consistency.

Time series with less than four years of daily records after quality control were discarded from PMET-obs. Due to large data gaps in the raw time series, lake level time series were only reconstructed when more than one station recorded a single lake, which was common in binational lakes. Given the growing demand for large-sample datasets, the upstream area corresponding to each stream gauge was delimited using NASADEM^66^ and several climatic and geographic attributes were derived following current guidelines for hydrological datasets^12^. The list of all catchment attributes and dataset sources can be found in Table 3.

**Table 3:** Climatic, hydrological, and geographic attributes calculated for each catchment as part of PMET-obs. The abbreviation corresponds to the attribute name in PMET-obs.

Abbreviation	Unit	Details	Reference
total_area	km^2^	Total catchment area calculated using NASADEM	NASA JPL^66^
total_area_camels	km^2^	Total catchment area obtained from CAMELS-CL	Alvarez-Garreton *et al*.^18^
int_area	km^2^	“Interstation” area (area of the station excluding, if needed, the catchment areas of nested upstream catchments)	NASA JPL^66^
elev_mean	m.a.s.l	Mean catchment elevation from NASADEM	NASA JPL^66^
elev_median	m.a.s.l	Median catchment elevation from NASADEM	NASA JPL^66^
slope_mean	deg	Mean catchment slope from NASADEM	NASA JPL^66^
lake_cover	%	Percentage of lake cover	Messager *et al*^120^.
forest_cover	%	Percentage of forest cover	Hansen *et al*^121^.
lai_max	—	Maximum monthly mean of leaf area index (LAI).	Mao and Yan^122^
lai_diff	—	Difference between the maximum and minimim monthly LAI	Mao and Yan^122^
glacier_cover	%	Glacier cover based on Randolph Glacier Inventory Version 6	RGI Consortium^123^
glacier_dhdt	mm	Storage change expressed as the average glacier mass balance	Hugonnet *et al*.^55^
Q_m3_s	m^3^ s^−1^	Mean annual streamflow (only calculated for stream gauges with more than 10 years of data)	PMET-obs
Qint_m3_s	m^3^ s^−1^	Mean “interstation” streamflow calculated from Q_m3_s	PMET-obs
Qint_mm_y	mm yr^−1^	Mean “interstation” specific streamflow calculated from “Q_m3_s” and “int_area”	PMET-obs
dam	[0,1]	Presence (dam = 1) or absence (dam = 0) of large dams.	PMET-obs
p_mean_PMET	mm yr^−1^	Mean annual precipitation calculated from PMET-sim (1980–2020)	PMET-sim
pet_mean_PMET	mm yr^−1^	Mean annual potential evaporation calculated from PMET-sim (1980–2020)	PMET-sim
aridity_PMET	—	Aridity index calculated using p_mean_PMET and pet_mean_PMET	PMET-sim
high_prec_freq_PMET	days	Frequency of high precipitation days, where precipitation ≥5 times mean daily precipitation	PMET-sim
high_prec_dur_PMET	days	Average duration of high precipitation events	PMET-sim
low_prec_freq_PMET	days	Frequency of low precipitation days, where precipitation <1 mm d^−1^	PMET-sim
low_prec_dur_PMET	days	Average duration of low precipitation events	PMET-sim
frac_snow_PMET	%	Fraction of precipitation falling as snow (threshold: 0 °C)	PMET-sim

### PMET-sim development

The development of PMET-sim consisted of three steps: i) the selection of the reference gridded product, ii) the downscaling of the selected product, and iii) the bias correction procedure (Fig. 2b). The reference gridded product provides a baseline for the correction in the following steps. The downscaling increases the spatial resolution (~0.5°) to a higher resolution of 0.05°, and the bias correction process addresses potential biases found in the selected reference gridded product.

#### Selection of the reference gridded product

Four gridded reanalysis datasets, which do not use *in situ* measurements from the study area, were evaluated to select the reference gridded product: ECMWF Reanalysis v5^67^ (ERA5), Modern-Era Retrospective analysis for Research and Applications Version 2^68^ (MERRA-2), Climate Forecast System Reanalysis^69^ (CFSR), and the RegCM4-CR2^70^ (CR2REG) (Table 2). The four datasets cover the entire latitudinal gradient of Western Patagonia (40–56°S), have a minimum resolution of 0.5°, and span a minimum period of 30 years. Previous validations have shown that reanalysis products outperform satellite estimates in mid and high latitudes due to their ability to represent large-scale frontal systems^14^.

The selection consisted of a point-to-pixel comparison between each gridded dataset and PMET-obs. In this method, monthly precipitation and air temperature values were compared against their corresponding grid cell values^71,72^. Precipitation performance was measured using the modified Kling-Gupta efficiency^73^, which disaggregates the overall performance into three components: linear correlation (r), bias ratio (β), and variability ratio (γ). On the other hand, the temperature performance was measured using the mean bias (β’), and the standard deviation ratio (γ’). As weather stations are typically located at low elevations (i.e., valleys), each gridded alternative was compared with a pseudo-corrected version of the PMET-obs temperature time series, which was modified based on the mean elevation of the corresponding grid-cell and the lapse rate used in the downscaling procedure (see next section). All indicators were calculated using the *hydroGOF* R package v0.4^74^. Considering that ERA5 achieved the best correlation for precipitation and a good representation of the annual temperature cycle (Supplementary Figure S1), we selected this alternative as the best gridded product for the next stages of development of PMET-sim.

#### Downscaling

To increase the spatial resolution to 0.05°, the downscaling procedure is applied to the selected gridded product (ERA5) and varied according to the variable. The temperature downscaling was based on the NASADEM^66^ digital elevation model, and a spatially and temporally constant environmental lapse rate of 6.5 °C km^−1^, a value commonly used in Western Patagonia^61^. Although recent studies in Patagonia have demonstrated the variability of this value^75^, preliminary results have shown that seasonal variation of this value does not significantly improves performance. Due to the lack of precipitation stations at high altitudes, precipitation downscaling was limited to a bilinear filter.

#### Bias correction

Once ERA5 was downscaled (hereafter ERA5d; Fig. 2b), the statistical bias correction process was performed. The bias correction of the maximum and minimum temperatures followed the mean and variance scaling method^76,77^ (Fig. 2b). This approach guarantees that the bias-corrected temperature time series of PMET-sim have the same mean and standard deviation (i.e., variance) as the ground-based time series of PMET-obs. Following the point-to-pixel comparison of the temperature, the simulated temperature obtained from ERA5d was compared with the pseudo-corrected version of PMET-obs, which takes into account the mean elevation of the grid-cell and the previously used lapse rate (6.5 °C km^−1^).

The approach is based on two parameters (β_T_ and α_T_), which scale the mean and variance of the temperature, respectively. In a first step, β_T_ is calculated as the difference between the mean (µ) of the simulated time series from ERA5d (T_S_) and the mean of the pseudo-corrected version of PMET-obs (T_o_) (Eq. 1).

1 $${{\rm{\beta }}}_{{\rm{T}}}={{\rm{\mu (T}}}_{{\rm{S}}}{\rm{)}}-{{\rm{\mu (T}}}_{{\rm{O}}}{\rm{)}}{\rm{.}}$$

Thereafter, the mean-corrected simulated time series (T_S_ - β_T_) is shifted to a zero mean (T_S1_, Eq. 2).

2 $${{\rm{T}}}_{{\rm{S1}}}=\left({{\rm{T}}}_{{\rm{S}}}-{{\rm{\beta }}}_{{\rm{T}}}\right)-{\rm{\mu }}\left({{\rm{T}}}_{{\rm{S}}}-{{\rm{\beta }}}_{{\rm{T}}}\right).$$

The variance scaling parameter (α_T_) was then calculated from the ratio between the standard deviations (σ) of T_o_ and T_S1_ (Eq. 3).

3 $${{\rm{\alpha }}}_{{\rm{T}}}=\frac{{\rm{\sigma }}\left({{\rm{T}}}_{{\rm{O}}}\right)}{{\rm{\sigma }}\left({{\rm{T}}}_{{\rm{S1}}}\right)}.$$

In a final step, the corrected temperature (T_S2_; Eq. 4) was obtained using the parameters β_T_ and α_T_ and T_S1_ of Eq. 2. To account for seasonal biases, the parameters β_T_ (Eq. 1) and α_T_ (Eq. 3) are calculated independently for each month.

4 $${{\rm{T}}}_{{\rm{S2}}}={{\rm{T}}}_{{\rm{S1}}}\cdot {{\rm{\alpha }}}_{{\rm{T}}}+{\rm{\mu }}\left({{\rm{T}}}_{{\rm{S}}}-{{\rm{\beta }}}_{{\rm{T}}}\right){\rm{.}}$$

The bias correction of PMET-sim precipitation followed a quantile mapping approach (Fig. 2b), which has been used in several meteorological datasets^78–80^. This method attempts to find a “transfer function” between the simulated (PP_s_) and observed (PP_o_; PMET-obs) cumulative distribution functions. In this case, the transfer function was based on a linear parametric transformation^81,82^ (Eq. 5).

5 $${{\rm{PP}}}_{{\rm{o}}}{}^{\ast }={{\rm{\alpha }}}_{{\rm{PP}}}+{{\rm{PP}}}_{{\rm{s}}}\cdot {{\rm{\beta }}}_{{\rm{PP}}}.$$

In Eq. 5, PP_o_* indicates the best estimate of PP_o_, and α_PP_ and β_PP_ are the monthly parameters subjected to calibration. Following Piani *et al*.^81^, the linear parametric transformation was fitted to the fraction of the cumulative distribution function (CDF) corresponding to observed wet days (PP_o_ > 0 mm d^−1^) by minimising the residual sum of squares. Other transformation functions, such as power and exponential, showed similar performance despite having more parameters. The bias correction was performed in the *qmap* R package v1.0^82,83^.

For the three variables (PP, T_max_ and T_min_), two parameters (α and β) were obtained for each time series and month (36 parameters in total). We used random forest (RF) regression models to derive regional gap-free maps for each parameter (Fig. 2b). RF regression models generate predictions using an adaptation of Leo Breiman’s RF algorithm, a supervised machine learning method^84,85^. These models have been successfully applied in several water resources studies using numerous climatic and geographic predictors^19,86,87^. In each RF model, 500 regression trees were used as an ensemble, with each tree having a minimum leaf size of five. At each split, two variables were randomly selected as candidates. From the full list of predictors (Table 4), we used backward selection (i.e., recursive feature elimination) with external validation to select the best predictors for each parameter and month. For external validation, we used leave-one-group-out cross-validation (LOGTCV) with 100 samples distributed across groups, with each group containing 90% of the data. Leaving out one group at a time and repeating the process for all groups provides a robust assessment of the model’s ability to generalise across groups. This procedure was performed using the R packages *randomForest* v4.7^88^ and *caret* v6.0^89^. Overall, the climate predictors, such as mean precipitation or temperature, were more important than geographic predictors (Supplementary Figure S2). To avoid abrupt discontinuities in the gridded precipitation, the resulting maps for α_PP_ and β_PP_ were subsequently smoothed with a Gaussian filter with a sigma size equal to the spatial resolution of PMET-sim (0.05°).

**Table 4:** Predictors used in random forest regression models.

Predictor	Product	Details	Reference
Elevation	NASADEM	Surface elevation (90 m)	NASA JPL^66^
Distance to coast	NASADEM	Logarithmic distance to the coast using NASADEM	NASA JPL^66^
Aspect	NASADEM	Calculated from NASADEM	NASA JPL^66^
West gradient	NASADEM	West component of the elevation gradient	NASA JPL^66^
Mean precipitation	ERA5	Mean raw annual precipitation (1980–2020)	Hersbach *et al*.^67^
Mean air temperature	ERA5	Mean raw annual temperature (1980–2020)	Hersbach *et al*.^67^
Aridity index	ERA5	Ratio between long-term annual precipitation and potential evaporation (Ep). Ep was derived from ERA5 using the Hargreaves equation (1980–2020)	Hersbach *et al*.^67^
Cloud cover	CLDCOV	Cloud cover frequency (2000–2014)	Wilson and Jetz^124^

All predictors were resampled to 0.05°.

Once the gridded parameters were derived from the RF procedure, we used the proposed bias correction methods to obtain the corrected values of precipitation, and maximum and minimum temperature for PMET-sim over 1980–2020. In addition, to correct precipitation undercatch, we followed the methodology proposed by Beck *et al*.^19^ (Fig. 2b), where the true long-term precipitation is inferred using the Budyko framework^90–92^. The Budyko framework^93^ is a parsimonious first-order empirical equation relating long-term precipitation (PP), potential evaporation (Ep), and actual evaporation (E) that assumes: long-term PP is the sum of long-term E and long-term runoff (R); long-term changes in water storage (ΔW) can be neglected; and PP is the only water input, and R and E are the only outputs^19^. To address these assumptions, we discarded catchments with dams (n = 5) and used the modified curve proposed by Liu *et al*.^94^ that includes glacier mass balance in the water balance:

6 $$\frac{{\rm{E}}}{{\rm{PP}}-\Delta {\rm{W}}}=1+\frac{{\rm{Ep}}}{{\rm{PP}}-\Delta {\rm{W}}}-{\left[1+{\left(\frac{{\rm{Ep}}}{{\rm{PP}}-\Delta {\rm{W}}}\right)}^{{\rm{W}}}\right]}^{\frac{1}{{\rm{W}}}},$$

where w is an empirical parameter representing catchment characteristics (unitless), and ΔW is the change in storage expressed as the average glacier mass balance in equivalent water column height (mm). We estimated the mass change for each catchment from geodetic mass balances for 2000–2019^55^. Note that Western Patagonia has a large native forest cover, low population density and many protected areas, so other implicit assumptions, such as the natural flow regime, can be reasonably assumed in the study area.

We first estimated long-term PP for each interstation catchment from long-term runoff (R) and potential evaporation (Ep). We then calculated interstation R for all catchments without dams in the PMET-obs dataset (n = 104). Long-term Ep was obtained directly from GLEAM v3.6a^95^, a process-based but semi-empirical model that calculates total evaporation and its individual components from satellite and reanalysis data (MSWX net radiation and air temperature). In preliminary stages, GLEAM 3.6a showed an adequate performance with respect to the ground-based data from the INIA institution (Table 1; Supplementary Figure S3). In both cases, long-term averages of R and Ep were calculated for the period 1980–2020. The empirical parameter w was estimated from previous results of the Chilean Water Balance for 1985–2015^58,59^. To date, this water balance is the largest hydrological modelling effort performed in Chile, including the binational catchments with Argentina. Specifically, we use the median w estimated for the selected catchments (w = 1.2), assuming that there are no significant differences between the two periods.

Once the true long-term precipitation was calculated, we calculated bias correction factors (BCFs) for each interstation catchment. Approximately 40% of the catchments had a BCF greater than 1.3, suggesting a significant underestimation of the precipitation necessary to generate the observed streamflows over the last decades (Supplementary Figure S4). The mean BCF obtained from the full dataset was 1.04 ± 0.45 ( ± 1 standard deviation). We then used RF regression models to derive regional gap-free BCF maps. Interstation regions with BCF > 2.5 (n = 2) were considered erroneous and were discarded from the training set (n = 102). Predictors were selected using backward selection and LOGTCV as external validation (Table 4). Based on this approach, the most important predictors of the RF regression were mean annual precipitation, elevation and aridity index (Supplementary Figure S2). The areas characterised by high BCFs were located on the western side of the Andes and were described by pronounced elevation gradients, high altitude and precipitation above 3,000 mm (Supplementary Figure S4). Following Beck *et al*.^19^, the BCFs were truncated at a lower bound of 1 because precipitation is more likely to be under than overestimated due to gauge undercatch^96,97^ and the low-elevation bias in gauge placement^98,99^. Finally, the BCFs were applied to the previously bias-corrected precipitation to obtain the final gridded PMET-sim precipitation.

## Data Records

The complete PatagoniaMet dataset (v1.1) can be found at: 10.5281/zenodo.7992760^100^.

### PMET-obs dataset

The quality-controlled data of each variable of PMET-obs are stored in separate.*csv* files with the following naming convention: *variable_PMETobs_timeperiod_version.csv*. Each variable has an additional.csv file with the metadata for each station (*variable_PMETobs_version_metadata.csv*). The metadata file contains the station name (*gauge_name*), the institution, the station location (*gauge_lat* and *gauge_lon*), the NASADEM elevation (*gauge_alt*) and the total number of daily records (*length*). In addition, the precipitation and temperature metadata include the number of monthly outliers (third step in the methods) and the number of changepoints (fourth step in the methods). In order to make transparent the possible erroneous data discarded from the quality-controlled version, a.zip file with the raw data of all variables is included in the dataset (*raw_data_PMETobs_version.zip*). Regarding the catchment dataset, the set of catchment boundaries is stored in shapefiles (each with a *gauge_id*), which are compiled into a.zip file (b*asins_PMETobs_version.zip*). The attributes calculated for each catchment can be found in the corresponding metadata file (*Q_PMETobs_version_metadata.csv;* Table 3). In addition, the file *Q_PMETobs_version_water_balance.csv* contains the water balance for the catchments that were part of the hydrological modeling validation (next section).

**Fig. 3:**
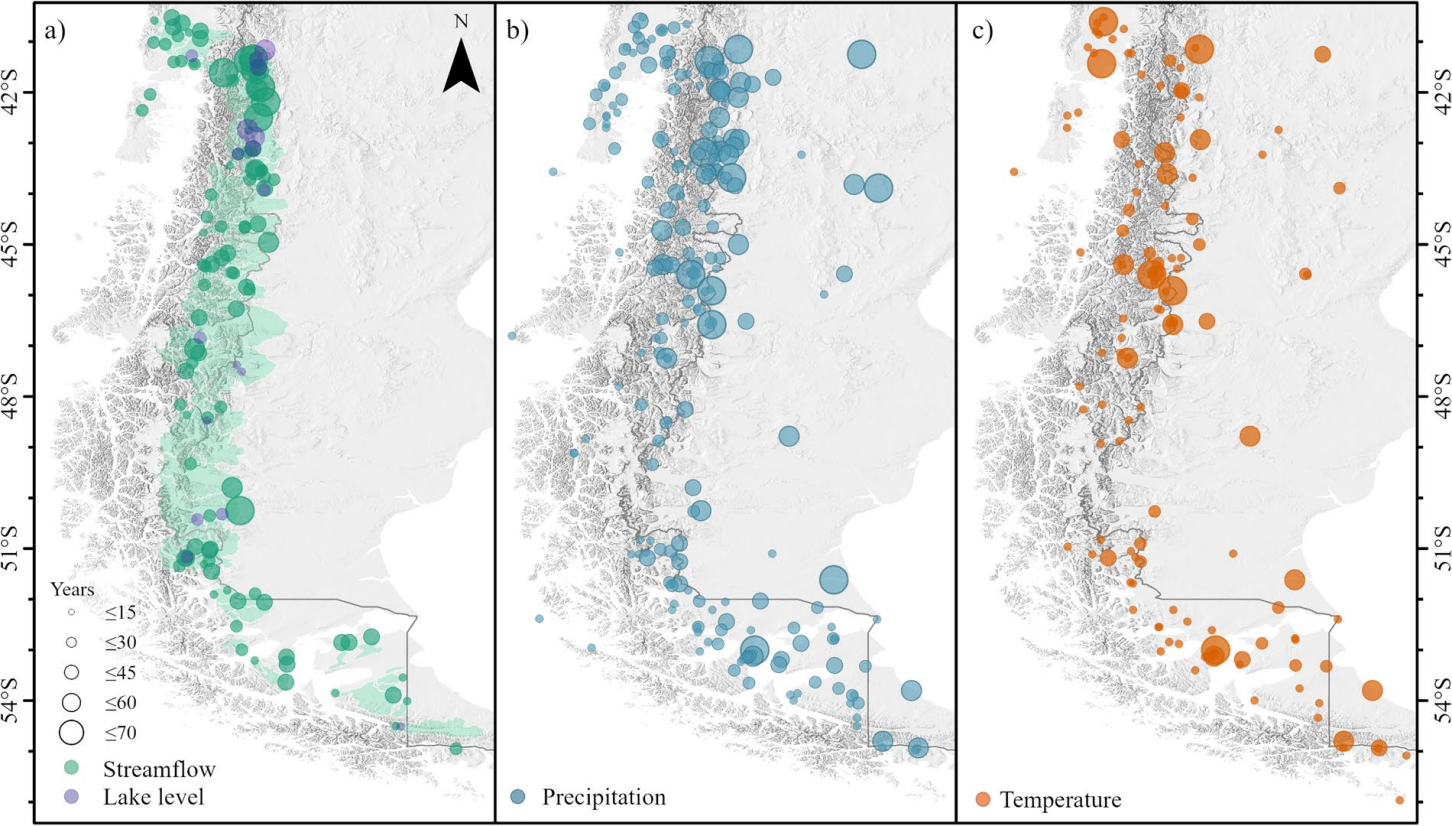
Spatial distribution of hydrometeorological stations in the PMET-obs dataset. (**a**) Stream gauges and lake level stations. (**b**) Precipitation. (**c**) Air temperature. The size of the circles is proportional to the number of years with records of each time series. The coloured areas in (**a**) indicate the catchments delimited by the stream gauges.

In summary, the PMET-obs dataset consists of 231, 129, 31, 109 and 23 time series of precipitation, air temperature, potential evaporation, stream gauges and lake levels, respectively (Fig. 3). Considering the area of Western Patagonia (~400,000 km^2^), the spatial density of precipitation, temperature and streamflow stations is one station per 1,700 km^2^, 3,100 km^2^ and 3,600 km^2^, respectively. The catchment area covered by all stream gauges is only one third of the total area of Western Patagonia (Fig. 3a). At the temporal scale, the median number of years (≥11 months of data) with precipitation, temperature and streamflow data is 18, 10 and 28 years, respectively. Only 17% of all time series have more than 30 years of data. Most stations with long-term records are located near major human settlements (e.g., Bariloche, Coyhaique and Punta Arenas; Fig. 1c) and near some rivers of hydroelectric interest (e.g., Puelo, Pascua and Baker; Fig. 1a).

### PMET-sim dataset

The gridded data of PMET-sim are stored in *netcdf* files with the following naming convention: *variable_PMETsim_timeperiod_version.nc*. All variables (precipitation and maximum and minimum temperature) have a spatial resolution of 0.05°, and cover the period 1980–2020.

**Fig. 4:**
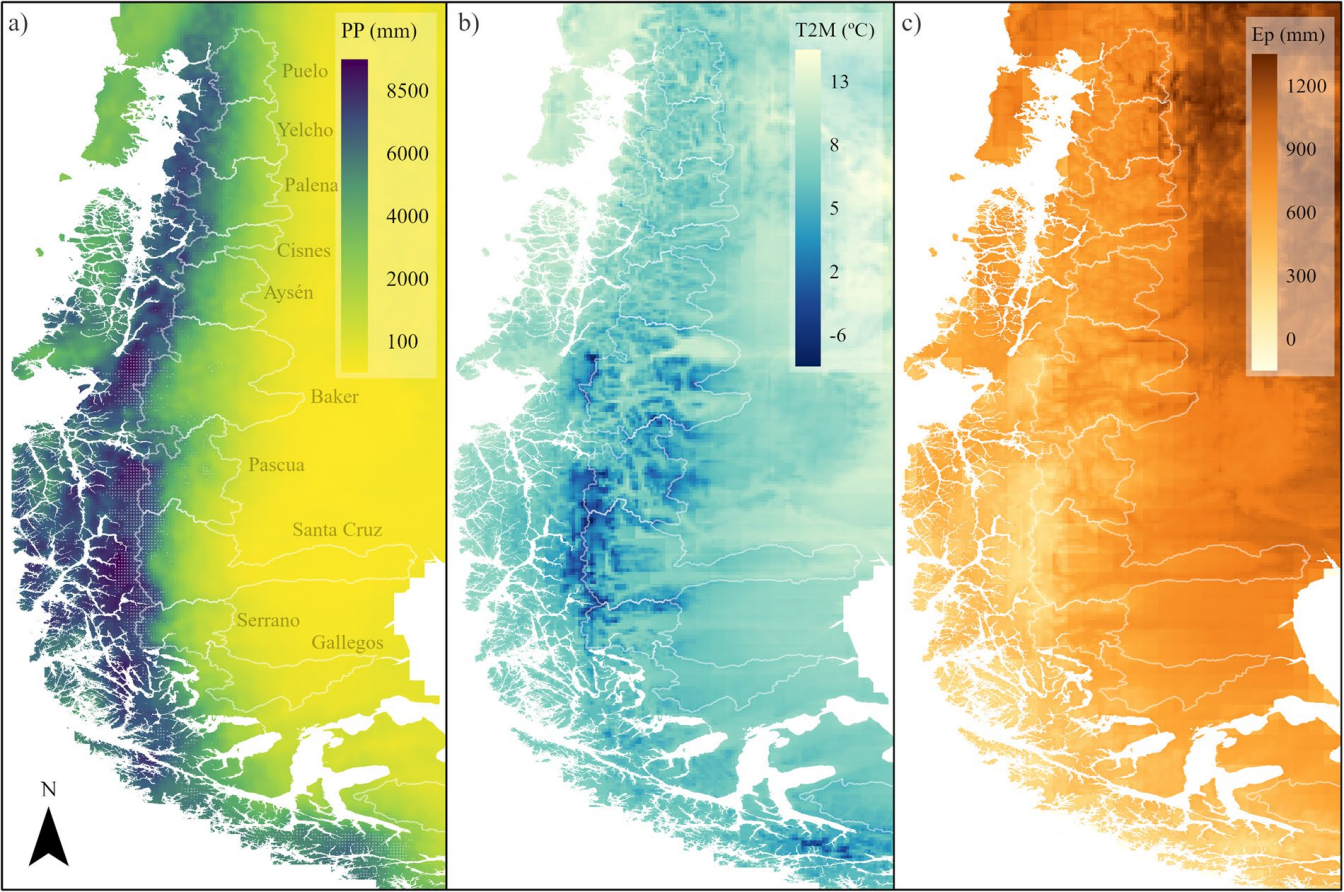
Long-term annual mean climate obtained from PMET-sim (1990–2020). (**a**) Precipitation (PP). (**b**) Air temperature (T2M). (**c**) Potential evaporation (Ep). Ep was calculated using the Hargreaves equation. Dotted areas in (**a**) show the glacier areas from RGI v6.0^123^. The white outlines indicate the main basins.

The PMET-sim spatial patterns of precipitation show a clear distinction between the western (>4,000 mm yr^−1^) and the eastern (<1,000 mm yr^−1^) side of the Andes (Fig. 4a). The maximum annual values were located in the Northern and Southern Patagonian Icefields (NPI and SPI) with mean values of 6,090 mm and 6,080 mm, respectively. These values are in agreement with Sauter^39^, who found that the icefield-wide precipitation averages (period 2010–2016) are likely to be within 5.38 ± 0.59 and 6.09 ± 0.64 m w.e. yr^−1^ on the NPI and 5.06 ± 0.51 and 5.99 ± 0.59 m w.e. yr^−1^ on the SPI according to the regional moisture flux. The catchments located in the northern area (Puelo to Cisnes) had a mean annual precipitation of 2100 mm, while the catchments located in the southeastern area had a mean annual precipitation lower than 500 mm (Gallegos and Río Grande). Most of the main catchments had mean annual temperatures between 3.0 °C and 7.0 °C (Fig. 4b). Considering the daily variation of air temperature and a melting threshold of 0 °C, the Pascua and Santa Cruz Rivers catchments had the highest annual snow accumulation amounts, with mean values of 500 mm and 285 mm, respectively (~25% of the total precipitation in both cases). The comparison between mean annual precipitation and potential evaporation (Fig. 4c) suggests that all major catchments are limited by available energy.

## Technical Validation

### Validation approach

The validation of PMET-sim consisted of a comparison with four gridded datasets using two approaches: a point-to-pixel comparison with PMET-obs (monthly precipitation and temperature), and a performance assessment by hydrological modelling (Fig. 2c). In the first case, the performance was measured using the metrics previously used in the selection of the reference gridded product (KGE, β’ and γ’), while the second approach used the performance obtained from the calibration of the TUWmodel^23^. For both approaches, a 10-fold cross-validation was added to avoid overestimating the performance achieved by PMET-sim. Each group (i.e., PMET-sim version) was developed using only 90% of all the stations, and the performance was measured in the remaining 10% of the stations.

The four benchmarks selected for the validation of PMET-sim were the Center for Climate and Resilience Research Meteorological dataset version 2.5 (CR2MET v2.5), Multi-Source Weighted-Ensemble Precipitation v2.8 (MSWEP) and W5E5 v2.0 (Table 2). These products have shown a good performance over the study area due to the use of local and/or regional data in their development (e.g., data from DGA, DMC and SMN, Table 1). CR2MET is currently a widely used reference dataset for PP and T2M in Chile^18,31,57,70^, including the National Water Balance in southern Chile^58,59^. MSWEP v2.8 is a multi-source precipitation-only product that merges gauge, satellite and reanalysis data to reduce temporal mismatches between satellite reanalysis estimates and gauge observations. Previous versions of MSWEP have recently outperformed other state-of-the-art precipitation products over Chile^101^. Precipitation data from MSWEP v2.8 were complemented with air temperature from Multi-Source Weather (MSWX), a bias-corrected compatible meteorological product. The W5E5 v2.0 dataset is part of the Inter-Sectoral Impact Model Intercomparison Project (ISIMIP3b) and merges local data with bias-corrected reanalysis data^102^ (WFDE5). The downscaled version of ERA5 (ERA5d) was also included in the comparison to measure the performance before the inclusion of PMET-obs in the bias correction procedure.

The hydrological modelling validation used the TUWmodel^23^, which is a daily conceptual rainfall-runoff model that follows the structure of the Hydrologiska Byråns Vattenbalansavdelning (HBV) model^103^. The model consists of three routines: a snow routine, a soil moisture routine, and a flow routing routine, which use precipitation, temperature and potential evaporation as input variables. The snow routine incorporates a temperature-index model to capture the accumulation and melting of snow^104^, assuming an empirical relationship between air temperatures and melt rates. In this approach, the melting is based on a degree-day factor (mm d^−1^ C^−1^) and a specific temperature threshold, while the calculation of snowfall and snow accumulation considers the temperatures at which snow and rain occur. The soil moisture routine accounts for changes in root zone moisture content caused by evapotranspiration and runoff generation. Estimation of actual evapotranspiration is based on potential evaporation. In addition, a model parameter is used to determine the soil moisture level at which actual evapotranspiration equals potential evaporation. Finally, the runoff routing module handles the movement of water across hillslopes and streams. It uses a runoff response function consisting of two reservoirs representing upper and lower storage zones. Runoff from the reservoirs in each elevation zone is aggregated and directed through a triangular transfer function for routing purposes. We selected the TUWmodel because of its extensive use in hydrological applications in snow-dominated catchments^105–108^.For example, Baez-Villanueva *et al*.^31^ used this model to calibrate a set of 100 near-natural catchments with a diverse hydroclimatic and geomorphological characteristics from the CAMELS-CL dataset, achieving a good overall performance (median KGE > 0.77).

The spin-up, calibration and validation periods of the hydrological modelling were 1987–1989 (3 years), 1990–2005 (16 years) and 2006–2020 (15 years), respectively. In contrast to central Chile, western Patagonia has not experienced consecutive dry years, and therefore both periods (calibration and validation) include dry and wet years. Based on the PMET-obs dataset, we selected 71 catchments with more than two-thirds of the records during the calibration period and no dams. Following the CemaNeige model^106^, each selected catchment was divided into equal-area elevation zones. The number of elevation zones (EZ) was defined as EZ = (H_max_ − H_min_)/300, where H is the elevation obtained from NASADEM (Table 4). If EZ > 5, EZ was set to 5 to be consistent with the spatial resolution of the different atmospheric forcings (≥0.05°) in mountainous areas. Based on the maximum and minimum daily temperature of each dataset, Ep was calculated using the Hargreaves equation and the PyEt package^109^. To maximise the KGE between observed and simulated streamflow, the automatic calibration was performed for each combination of atmospheric forcing (n = 6) and each independent catchment using the *hydroPSO* R package v0.5^74,110^. *HydroPSO* is a global optimization R package that implements a state-of-the-art version of the Particle Swarm Optimization (PSO). This algorithm has been successfully used in several hydrological modelling applications^31,111,112^. The parameter ranges of the TUWmodel were based on Parajka *et al*.^106^ (Supplementary Table S1).

### Validation results

**Fig. 5:**
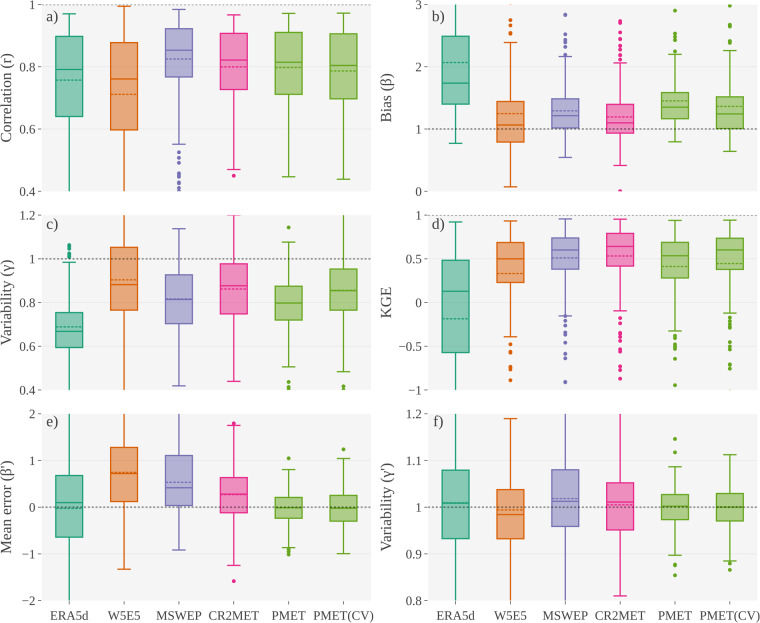
Performance metrics of precipitation (**a**–**d**) and air temperature (**e,f**) for ERA5d (downscaled version of ERA5), W5E5 v2.0, MSWEP v2.8 (WSWX for air temperature), CR2MET v2.5 and PMET-sim. The PMET-sim (CV) corresponds to the summarized results (“unseen” stations) from the 10-fold cross-validation. The different performance metrics were obtained using a point-to-pixel analysis using PMET-obs as the reference (period 1990–2020). The dotted line in each box plot represents the mean value. The horizontal dotted line in each panel represents the optimal value.

The development of PMET-sim improved several metrics with respect to ground-based observations (Fig. 5). For precipitation, PMET-sim achieved correlation values similar to ERA5d (Fig. 5a). MSWEP v2.8 reached the best correlation (median value = 0.87), while W5E5 showed the worst. PMET-sim reduced the median precipitation bias (β) from 2.1 in ERA5d to 1.3 (Fig. 5b), which represents an overestimation of 30% with respect to observations from meteorological stations (usually unshielded tipping bucket rain gauges). All models underestimate precipitation variability (Fig. 5c). CR2MET and W5E5 showed better median values (γ), but W5E5 presented a higher spread. CR2MET showed the best overall precipitation performance expressed by the KGE (Fig. 5d). Compared to ERA5d and PMET-sim, CR2MET showed a better KGE due to lower biases and a better reproduction of the precipitation variability. The temperature bias correction reduced the bias spread (standard deviation of β’) from 1.0 °C in ERA5d to 0.4 °C in PMET-sim (Fig. 5e). W5E5, CR2MET and MSWEP showed a warm bias lower than 1 °C. All models were able to reproduce the variability of the annual cycle expressed by γ’, with median values close to 1.0 in all cases (Fig. 5e). The results of the 10-fold cross-validation achieved similar values to the PMET-sim version developed with all available data (Fig. 5), which could be attributed to the fact that the predictors in the RF spatial regression were previously selected using LOGTCV as an external validation.

**Fig. 6:**
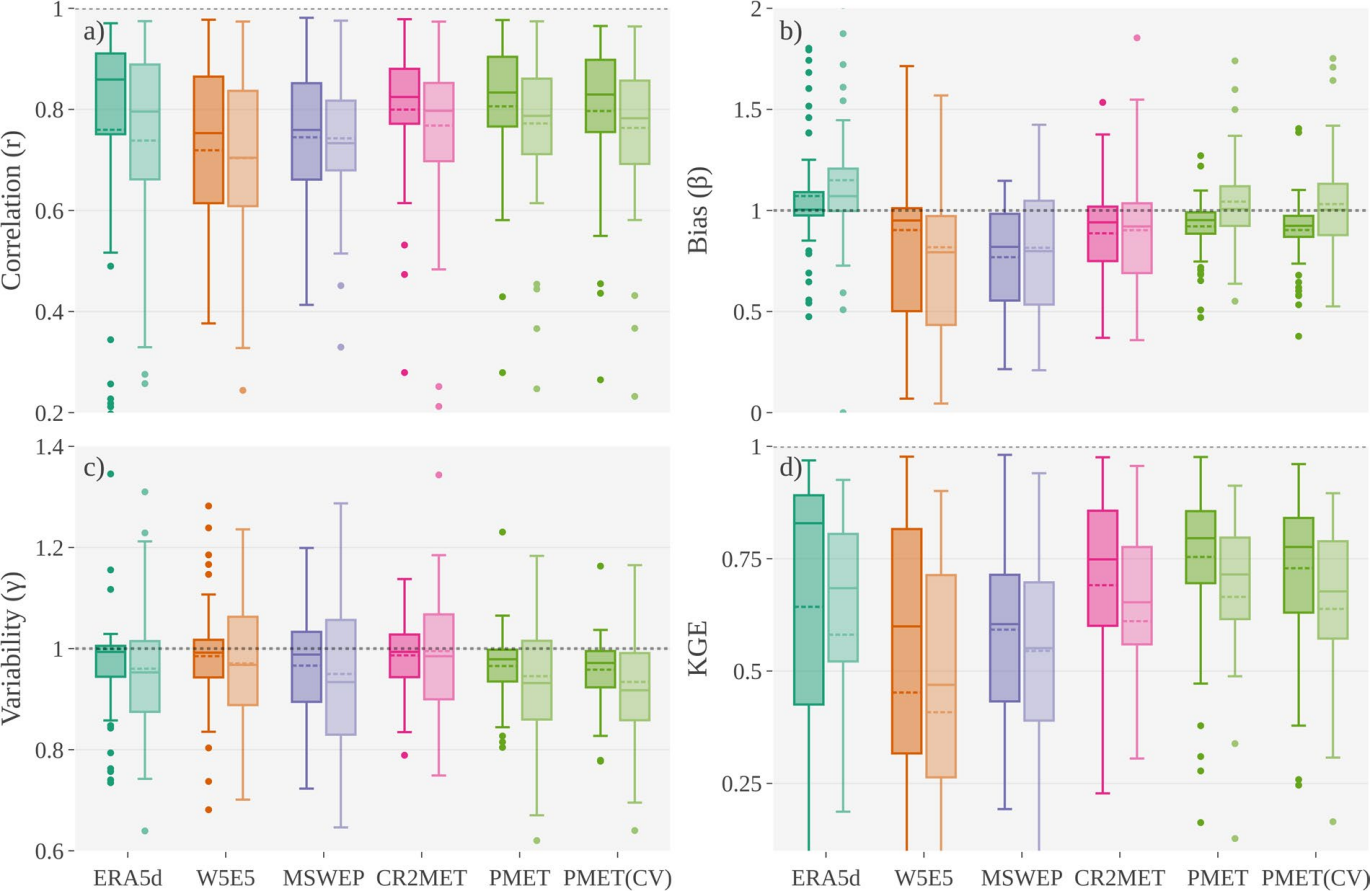
Hydrological model performance (on a monthly basis) under different atmospheric alternatives (ERA5d, MSWEP v2.8/MSWX, CR2MET v2.5, W5E5 v2.0 and PMET-sim). The PMET-sim (CV) corresponds to the summarized results (“unseen” stations) from the 10-fold cross-validation. The calibration (dark colours) and validation (light colours) periods were 1990–2005 and 2006–2020, respectively. The horizontal dotted line in each panel represents the optimal value.

Good performance obtained from the comparison with meteorological stations did not always translate into a good hydrological performance (Fig. 6). All forcing datasets showed median correlations values greater than 0.7 during the calibration and validation stages (Fig. 6a), with PMET-sim, ERA5d and CR2MET being the best forcing datasets (r > 0.8 during calibration). The bias (β) was the metric that showed the most variability among the forcing datasets. Despite the high biases of ERA5d and PMET-sim in Fig. 5b,both models achieved biases close to the optimum with a small spread (Fig. 6b). In terms of variability (γ), most datasets slightly underestimated the ground-based observations (Fig. 6c). Considering the performance during the calibration phase, PMET-sim achieved KGEs greater than 0.7 in 72% of the catchments compared to CR2MET, MSWEP and W5E5, which achieved 53%, 29% and 38% in the calibration period, respectively (Fig. 6d). In all metrics, the performance achieved in the “unseen” catchments of the 10-fold cross-validation was similar to that achieved with all available data.

## Usage Notes

This regional dataset contributes to the hydrological and atmospheric sciences by providing a novel dataset for Western Patagonia, which will improve data availability^113^ and research reproducibility^114^, and can be used to advance our understanding of the effects of climate change in this unique water reservoir for South America. Although the time series available in PMET-obs represent a clear advance for a variety of scientific applications, it is important to note that the density of precipitation stations (Fig. 3b) is still seven times lower than the one recommended by the WMO^115^ for mountainous areas (1 per 250 km^2^). On the other hand, the density of stream gauges is almost four times lower than recommended (1 per 1,000 km^2^). The best spatial density of gauging stations is found in the vicinity of the main human settlements (where different institutions measure the same meteorological variable), while in the western fjord zone there are large regions with few or no observations (Fig. 3). Taking this into account, it is important to note that there are large areas in PMET-sim without local validation. Nevertheless, PMET currently performs better hydrologically than any other regional and global gridded product available to date.

PatagoniaMet is envisioned as an open collaborative dataset that will be regularly updated with new records, incorporating additional meteorological variables, institutions and time-steps as they become available. This will provide a foundation for future hydrometeorological studies in Western Patagonia, which can be accessed and reviewed by anyone in the community.

### Supplementary information

Supplementary information

## Data Availability

The complete repository can be found at: https://github.com/rodaguayo/PatagoniaMet^116^.
