# Supplementary material for: PatagoniaMet: A multi-source hydrometeorological dataset for Western Patagonia
Source: Sci Data. 2024 Jan 2;11:6. doi: 10.1038/s41597-023-02828-2 (PMC10761917; doi:10.1038/s41597-023-02828-2)
Supplement: Supplementary file 1 — Supplementary information [file 41597_2023_2828_MOESM1_ESM.pdf]

Supplementary material for: “PatagoniaMet: A multi-source hydrometeorological dataset for Western Patagonia”

Rodrigo Aguayo[1,*], Jorge León-Muñoz[2,3], Mauricio Aguayo[1], Oscar Manuel Baez-Villanueva[4], Mauricio Zambrano-Bigiarini[5,6], Alfonso Fernández[7,8], Martin Jacques-Coper[6,9,10]

1. Facultad de Ciencias Ambientales, Centro EULA-Chile, Universidad de Concepción, Concepción, Chile.
2. Departamento de Química Ambiental, Universidad Católica de la Santísima Concepción, Concepción, Chile.
3. Centro Interdisciplinario para la Investigación Acuícola (INCAR), Concepción-Puerto Montt, Chile.
4. Hydro-Climate Extremes Lab (H-CEL), Ghent University, Ghent, Belgium
5. Departamento de Ingeniería Civil, Universidad de La Frontera, Temuco, Chile
6. Center for Climate and Resilience Research (CR2), Santiago, Chile
7. Departamento de Geografía, Mountain GeoScience Group, Universidad de Concepción, Concepción, Chile
8. Programa Ciencia Interdisciplinaria para las Montañas de los Andes del Sur (CIMASur), Universidad de Concepción, Concepción, Chile
9. Departamento de Geofísica, Universidad de Concepción, Concepción, Chile
10. Center for Oceanographic Research COPAS-Coastal, Universidad de Concepción, Concepción, Chile

*corresponding author: Rodrigo Aguayo (rodaguayo@udec.cl)

Table of contents:

- Figure S1: Selection of the reference gridded product
- Figure S2: Importance of random forest predictors of each parameter
- Figure S3: Performance metrics of potential evaporation (Ep) from GLEAM v3.6a.
- Figure S4: Bias correction factors (BCF) obtained from the Budyko framework.
- Table S1: Range of the parameters used during the calibration of the TUWmodel.

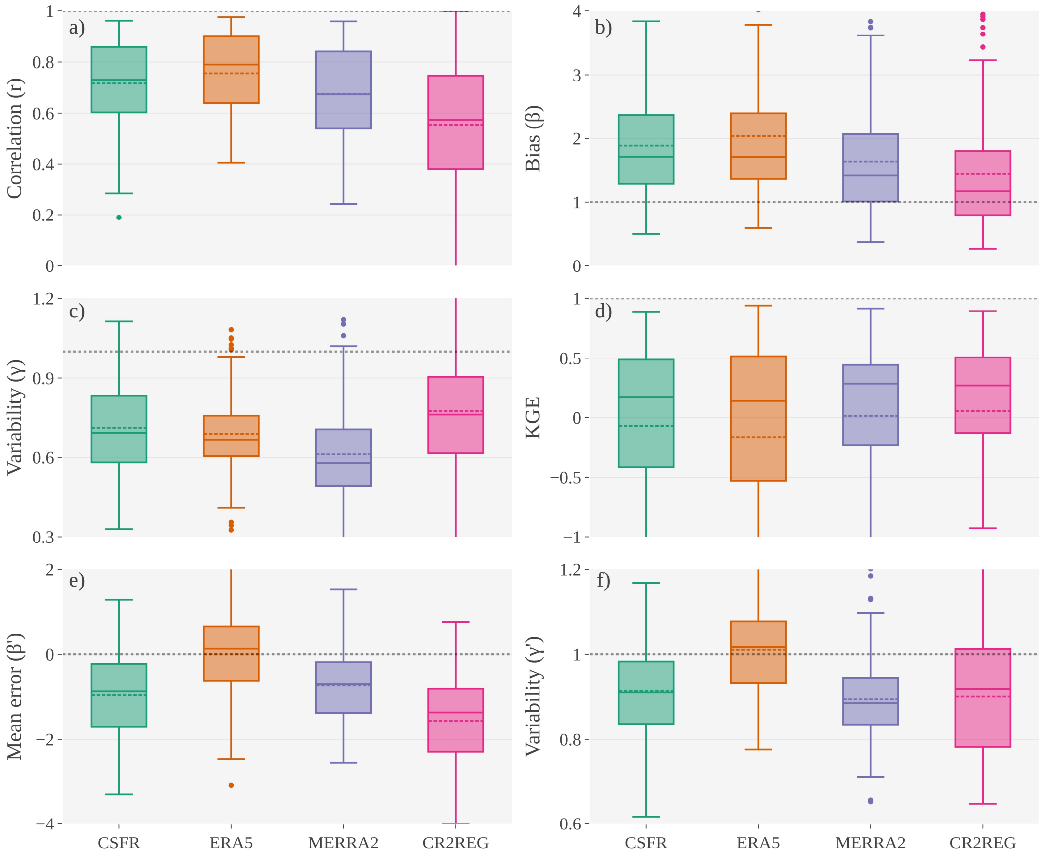

Figure S1. Performance of precipitation (a-d) and air temperature (e, f) for ERA5, MERRA2, CSFR and REGCR2 (1950-2019). The different performance metrics were obtained from a point-to-pixel analysis. The time step of the analysis was monthly. The horizontal dotted line in each panel represents the optimal value.

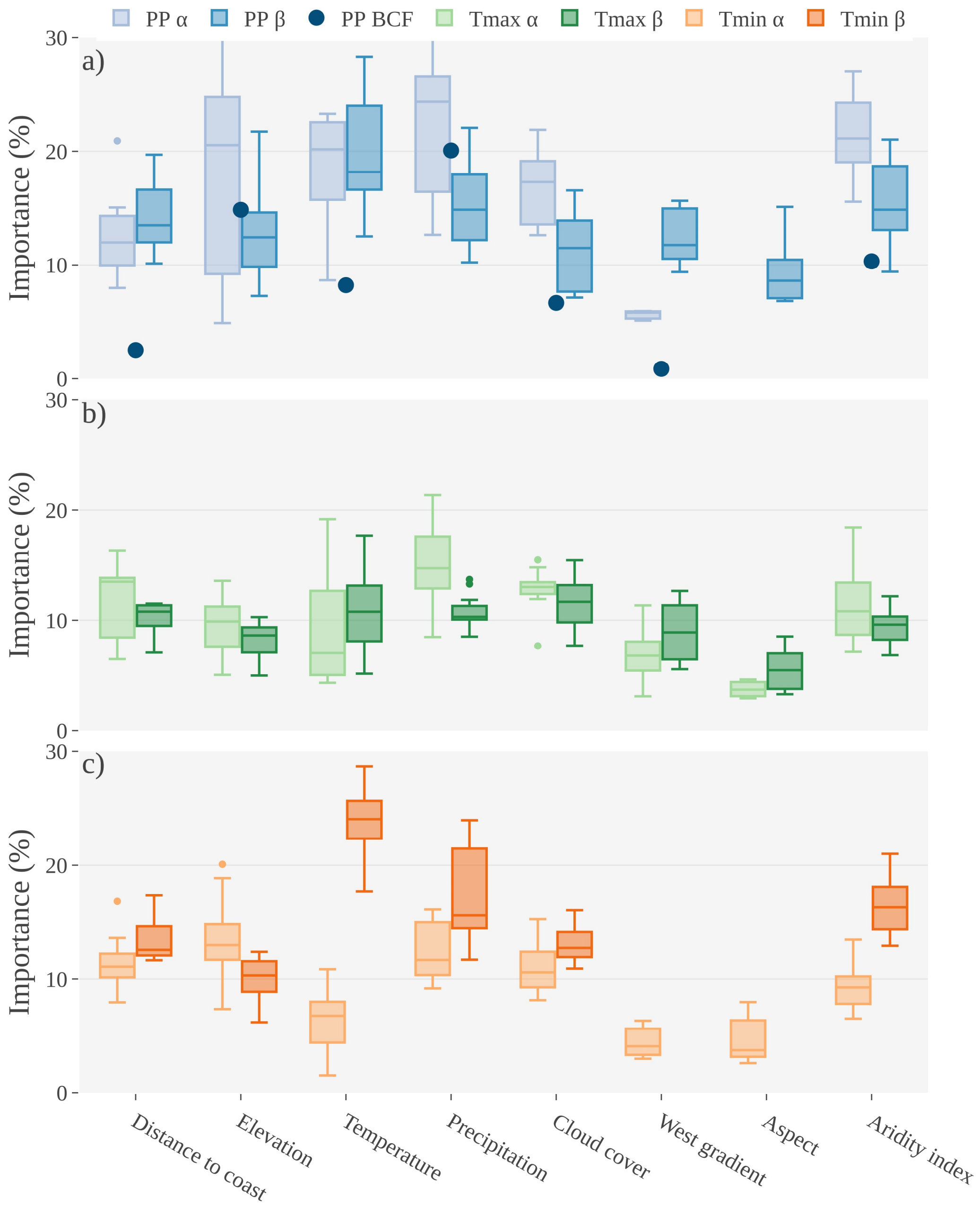

Figure S2. Importance of the random forest predictors for each parameter. Importance was represented as the increase (in %) in the mean squared error (MSE) on the out-of-bag samples when the variable (predictor) was excluded from the model.

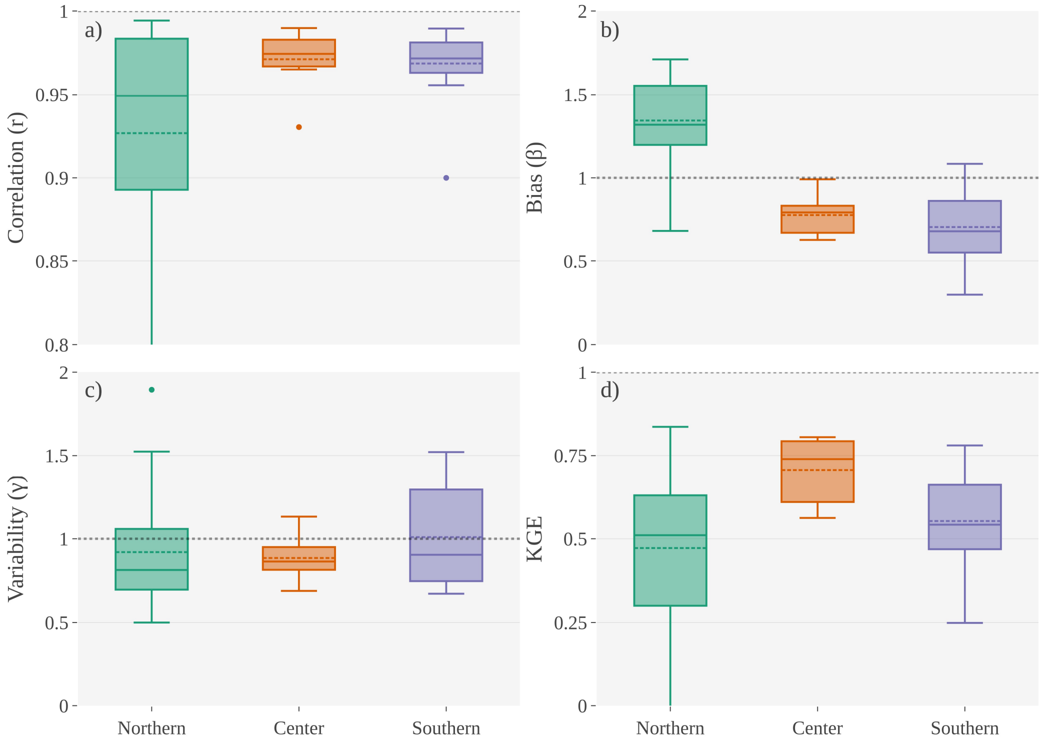

Figure S3. Performance metrics of potential evaporation (Ep) from GLEAM v3.6a. Zones are defined in Fig. 1. The metrics were obtained by comparing monthly simulations and observations at the corresponding grid cell. The horizontal dotted line in each panel represents the optimal value.

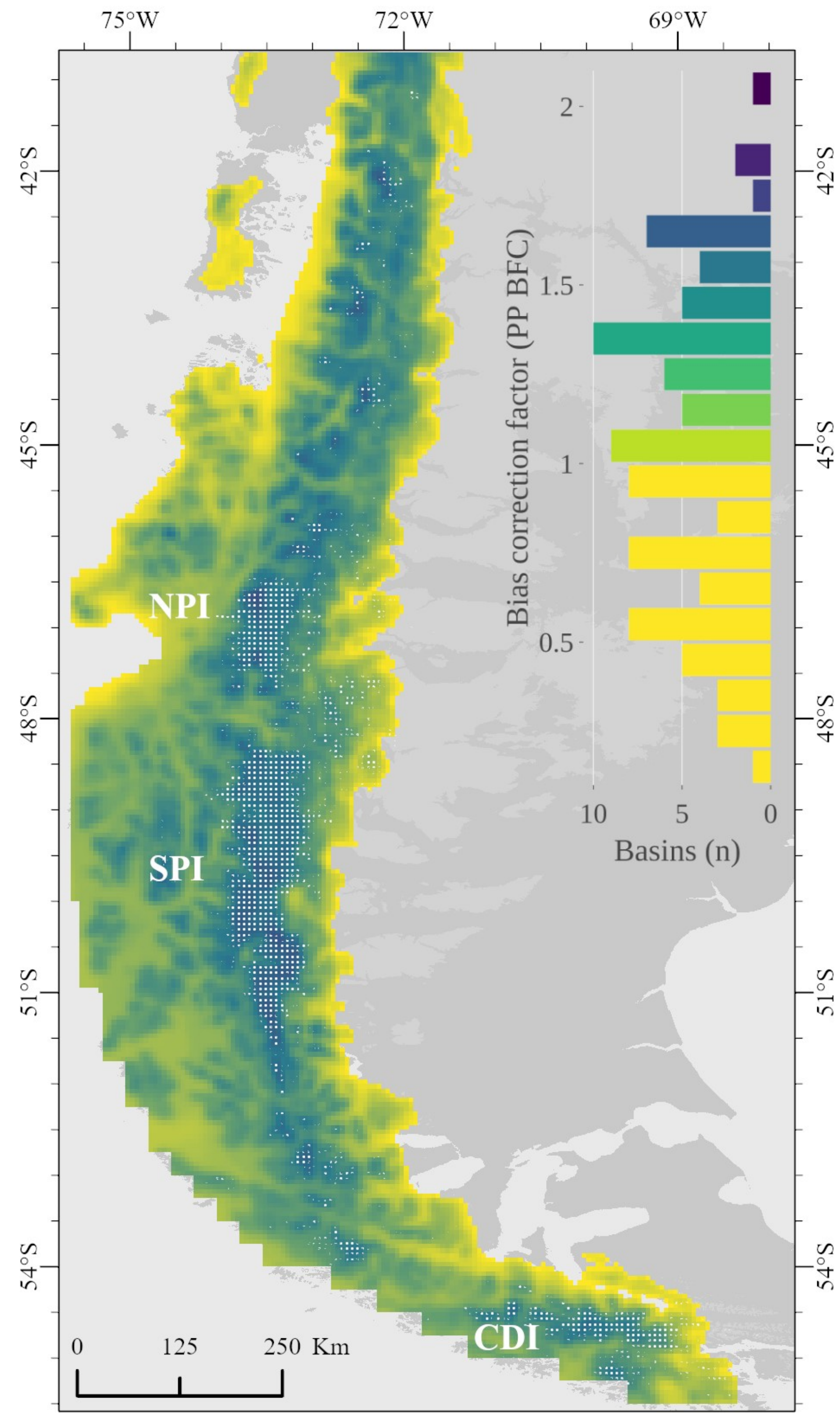

Figure S4. Bias correction factors (BCF) obtained from the Budyko framework. The histograms show the basins used by the random forest regression model. The BCF was calculated as the ratio between the true long-term precipitation and the uncorrected mean of PMET-sim mean. Dotted areas indicate glacier areas from the Randolph Glacier Inventory v6.0[117].

Table S1. Range of the parameters used during the calibration of the TUWmodel.

Parameter	Description	Units	Process	Range
SCF	Snow correction factor	-	Snow	Set to 1.0
DDF	Degree-day factor	mm / ºC / day	Snow	3.0 – 40.0
Tr	Temperature threshold for rain	ºC	Snow	1.0 – 6.0
Ts	Temperature threshold for is snow	ºC	Snow	-5.0 – 1.0
Tm	Temperature threshold above which melt starts	ºC	Snow	-2.0 – 2.0
LPrat	Limit for potential evaporation	-	Evaporation	0.1 – 1.0
FC	Field capacity	mm	Infiltration	10.0 - 3000
BETA	Non-linear parameter for runoff production	-	Infiltration	0.0 - 20.0
cperc	Constant percolation rate	mm / day	Infiltration	0.0 – 8.0
k0	Storage coefficient for very fast response	day	Runoff	0.0 – 1.5
k1	Storage coefficient for fast response	day	Runoff	1.5 – 6.0
k2	Storage coefficient for slow response	day	Runoff	6.0 – 18.0
lsuz	Threshold storage state	mm	Runoff	1.0 – 50.0
bmax	Maximum base at low flows	day	Runoff	0.0 – 30.0
croute	Free scaling parameter	day^{2} / mm	Runoff	10.0 – 40.0
